# Clinical characteristics and surgical treatment comparison of multisegmental spinal tuberculosis: a retrospective analysis

**DOI:** 10.3389/fmed.2025.1541745

**Published:** 2025-02-19

**Authors:** Zongqiang Yang, Zhangui Gu, Qiang Liu, Long Ma, Le Fei, Ningkui Niu, Jiandang Shi

**Affiliations:** ^1^Department of Orthopedic, General Hospital of Ningxia Medical University, Yinchuan, China; ^2^First Clinical Medical College, Ningxia Medical University, Yinchuan, China; ^3^Bone and Joint Tuberculosis Prevention and Control Research Center, General Hospital of Ningxia Medical University, Yinchuan, China

**Keywords:** multisegmental spinal tuberculosis, clinical characteristics, surgical treatment, efficacy evaluation, management

## Abstract

**Background:**

To analyze the therapeutic efficacy of intervertebral surgery in the treatment of multisegmental spinal tuberculosis (MSTB) by evaluating its clinical outcomes and perioperative indicators, thereby providing evidence-based insights to optimize surgical strategies and improve clinical decision-making.

**Methods:**

This retrospective cohort study analyzed 134 MSTB patients treated at our hospital between January 2000 and June 2022. Based on the surgical approach, patients were divided into two groups: Group A (*n* = 75), who underwent intervertebral surgery, and Group B (*n* = 59), who received non-intervertebral surgery. All patients underwent radical debridement, bone graft fusion, spinal canal decompression, and internal fixation to restore spinal alignment. To compare the clinical outcomes of these two surgical approaches, we assessed perioperative parameters, radiographic outcomes, laboratory inflammatory markers, neurological recovery, and complication rates. Statistical analyses included *t*-tests or Mann–Whitney *U* tests for continuous variables and chi-square or Fisher’s exact tests for categorical variables.

**Results:**

The mean follow-up duration was 63.24 ± 9.16 months for Group A and 64.05 ± 9.74 months for Group B. Group A demonstrated significantly shorter operative time and reduced intraoperative blood loss compared to Group B (*p* < 0.05). No significant differences were observed between the groups regarding preoperative, 6-month postoperative, and final follow-up values of erythrocyte sedimentation rate (ESR), C-reactive protein (CRP) levels, Cobb angle, or visual analog scale (VAS) scores (*p* > 0.05). Bone fusion rates were comparable between the two groups at 6 months, 1 year postoperatively, and at final follow-up, with complete fusion achieved in all patients (*p* > 0.05). The incidence of postoperative complications and neurological recovery rates did not differ significantly between the two groups (*p* > 0.05).

**Conclusion:**

Both intervertebral and non-intervertebral surgical strategies for MSTB can effectively restore spinal alignment and achieve satisfactory neurological recovery, provided that strict surgical indications are adhered to. Intervertebral surgery, with its shorter operative time and lower intraoperative blood loss, may offer additional perioperative advantages and serve as a viable option for MSTB management.

## 1 Introduction

Tuberculosis (TB), caused by *Mycobacterium tuberculosis* (Mtb), remains a major global health concern, with 8.2 million newly diagnosed cases and approximately 1.2 million deaths reported worldwide in 2023, imposing a substantial economic and social burden, particularly in developing countries (1). Spinal tuberculosis (STB) is the most common form of osteoarticular TB, accounting for 50–60% of all cases (2). It primarily affects the thoracic and lumbar spine (3), and while most patients present with single-segment lesions, the incidence of multisegmental spinal tuberculosis (MSTB), involving three or more vertebral segments, is rising due to delayed diagnosis and rapid disease progression (4).

Anti-tuberculosis chemotherapy forms the cornerstone of STB treatment, but surgical intervention is crucial for improving outcomes and managing complications such as spinal deformity, paravertebral abscesses, instability, and paraplegia (5). MSTB patients, due to extensive spinal involvement, face a higher risk of chronic pain, progressive deformity, and neurological impairment, with conservative management often failing to provide adequate disease control (6). Consequently, surgical strategies, including anterior-only, posterior-only, and combined anterior-posterior approaches, are widely employed. However, the optimal surgical approach for MSTB remains controversial, particularly concerning the extent of lesion debridement and fixation segment selection (7, 8).

Our research team has previously demonstrated the safety and efficacy of interbody fixation in single-segment thoracolumbar tuberculosis (9, 10). Preliminary investigations into its application for discontinuous MSTB have yielded promising results, but these studies were limited by small sample sizes and the absence of standardized criteria for fixation (11). Given the rising prevalence of MSTB and the lack of consensus on surgical management, this study aims to address these gaps. By analyzing clinical characteristics and comparing the efficacy of interbody fixation versus non-interbody fixation techniques, we seek to provide robust evidence to guide surgical strategies for MSTB and improve clinical outcomes.

## 2 Methods

### 2.1 General information

We conducted a retrospective analysis of 1,076 patients with STB treated in our department between January 2000 and June 2022. The inclusion criteria were: (1) confirmed diagnosis of STB based on at least one of the following—culture of *Mycobacterium tuberculosis* from biopsy or surgical specimens, histopathological evidence (acid-fast staining), metagenomic next-generation sequencing (mNGS), or a positive Xpert MTB/RIF test; (2) involvement of three or more vertebrae; (3) clear surgical indications; (4) no severe hepatic or renal insufficiency, with patients deemed fit for surgery; and (5) complete medical records, including hospitalization and follow-up data. Among the total cohort, 134 patients (12.45%) met the criteria for MSTB and were included in this study. Comprehensive evaluations were performed, covering clinical epidemiological characteristics, symptoms, laboratory findings, imaging features, and lesion segment distribution. All included patients underwent extensive debridement, spinal fusion, spinal canal decompression, and internal fixation for spinal alignment correction. Additionally, all patients were screened for HIV prior to surgery.

**Figure 1:**
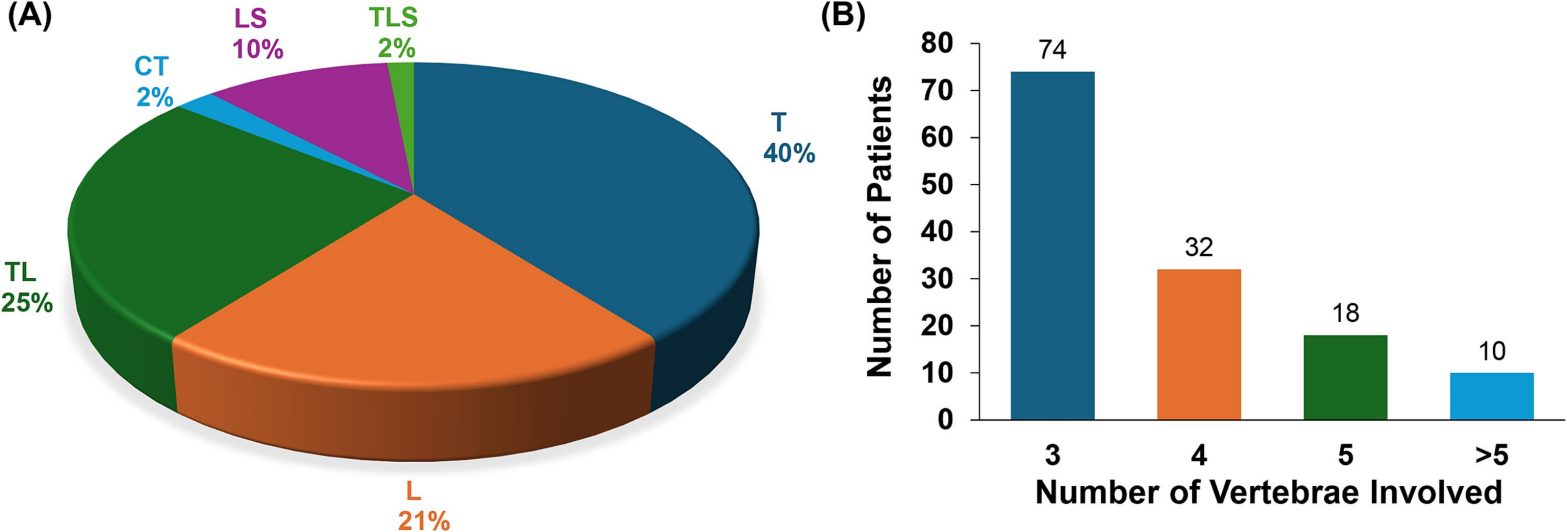
Distribution and number of vertebral segments in 134 patients with multisegmental spinal tuberculosis. **(A)** Distribution of vertebral segments involved in the lesions. **(B)** Number of vertebral bodies affected by the lesions. T, thoracic vertebra; L, lumbar vertebra; TL, thoracolumbar vertebra; CT, cervicothoracic vertebra; LS, lumbosacral vertebra; TLS, thoracolumbosacral vertebra.

The clinical and epidemiological characteristics of the 134 patients with MSTB are presented in Table 1. Laboratory test results and imaging features are displayed in Table 2, while the segmental distribution of lesions is shown in Figure 1. Baseline data for the two surgical groups are summarized in Table 3.

**Table 1:** Epidemiology and clinical symptoms.

Characteristics	Number	Mean ± SD or NO/%
General information		
Sex		
Male	65	48.51
Female	69	51.49
Age (years)	—	41.64 ± 16.80 (11–78)
Profession		
Farmer	88	65.67
Student	26	19.40
Staff	20	14.93
Diagnosis and treatment information		
Clinical characteristics		
Duration of disease (months)	—	19.71 ± 25.67
Fatigue	49	
Fever	38	28.36
Night sweats	51	38.06
Weight loss	21	15.67
Spinal pain	127	94.78
Hypoesthesia	8	5.97
Radiculalgia	18	13.43
Motor deficit	9	6.72
Pulmonary tuberculosis	67	50.00
Diabetes	4	2.99
HIV	0	—
Frankel grading		
A	0	0.00
B	3	2.23
C	27	20.15
D	52	38.81
E	52	38.81
Postoperative chemotherapy regimen		
Standard chemotherapy regimen	71	52.99
Ultra short course chemotherapy protocol	63	47.01

**Table 2:** Laboratory examination and imaging features.

Characteristics	Number	Percentage (%)
Laboratory inspection		
Anemia	21	15.67
Elevated sedimentation rate (2–43)	47	35.07
Elevated CRP (0–2.83)	128	95.52
Imaging characteristics		
Kyphosis	25	18.66
Scoliosis	3	2.24
Paravertebral abscess	73	54.48
Unilateral	55	41.05
Bilateral	18	13.43

**Table 3:** Comparison of general baseline data between Groups A and B.

Characteristics	Group A (*n* = 75)	Group B (*n* = 59)	Test value (*t*/*U*/*χ*^2^)	*p*
Age (years)	41.79 ± 18.19	41.21 ± 15.12	0.19	0.85
Male/female	33/42	32/27	1.39	0.24
Duration of disease (months)	21.05 ± 27.47	18.00 ± 23.31	0.68	0.50
ESR (mm/h)	39.06 ± 25.07	38.66 ± 23.39	0.09	0.93
CRP (mg/L)	29.34 ± 33.47	26.71 ± 20.33	0.53	0.60
VAS score (points)	7.25 ± 0.92	7.39 ± 1.00	−0.83	0.41
Number of affected vertebrae				
3	39	35	0.72	0.40
4	17	15	0.31	0.58
5	11	5	1.20	0.27
>5	8	4	0.23	0.63
Surgical approach				
Anterior-only approach	6	5	0.00	1.00
Posterior-only approach	11	2	3.60	0.06
Posterior-anterior approach	58	52	2.62	0.10

### 2.2 Preoperative preparation

Patients in both groups received strict bed rest and preoperative antitubercular therapy, which consisted of a combination of isoniazid (5 mg/kg/day, with a maximum dose of 300 mg), rifampicin (10 mg/kg/day, with a maximum dose of 0.6 g), pyrazinamide (25 mg/kg/day, with a maximum dose of 2.0 g), ethambutol (15 mg/kg/day, with a maximum dose of 1.6 g) and streptomycin [before 2010, (20 mg/kg/day, with a maximum dose of 1.0 g, was given by intramuscular injection, once per day)] for 2–3 weeks or longer until symptoms of systemic tuberculosis toxicity were resolved. Perioperative care included correction of hypoproteinemia, nutritional optimization, and ensuring hemoglobin levels exceeded 100 g/L while maintaining normal liver and renal function. Patients with comorbid conditions received appropriate management to eliminate surgical contraindications.

### 2.3 Surgical grouping and methods

#### 2.3.1 Surgical grouping

Patients were divided into two groups based on the extent of lesion removal and fusion, as well as the length of internal fixation: Group A (intervertebral surgery group): thorough lesion removal and fusion at the intervertebral level, with internal fixation limited to the intervertebral segments. Group B (non-intervertebral surgery group): thorough lesion removal and fusion at the intervertebral level, with internal fixation extending to one or more vertebrae above and below the lesion.

#### 2.3.2 Surgical approach

Both groups underwent comprehensive lesion debridement, spinal canal decompression, autologous bone graft fusion, deformity correction, and internal fixation. The surgical approaches included the anterior-only approach, posterior-only approach, and a combined posterior-anterior approach.

##### 2.3.2.1 Intervertebral surgical approach

###### 2.3.2.1.1 Anterior-only approach

Depending on the lesion’s location, the transthoracic, extrapleural, thoracoabdominal, extraperitoneal, nephrectomy, retroperitoneal “inverted eight,” or oblique lateral interbody fusion (OLIF) approaches were employed. Under general anesthesia, the patient was positioned laterally, and the lesion was precisely localized preoperatively. The skin, subcutaneous tissue, muscle, and fascia were incised to expose the lateral aspect of the spinal column, including the upper and lower margins of the affected vertebrae. Debridement was performed following established protocols, followed by spinal canal decompression, intervertebral fusion using autologous bone grafts, and anterior intervertebral fixation of the affected vertebrae.

###### 2.3.2.1.2 Posterior-only approach

Under general anesthesia, the patient was placed in the prone position, and the lesion was localized preoperatively. A midline posterior incision was made to expose the spinous process, lamina, and transverse processes of the affected vertebrae.

###### 2.3.2.1.3 Thoracic tuberculosis

Pedicle screws were inserted, and the spinous process, lamina, articular processes, transverse processes, and 3 cm of the affected-side rib were excised to expose the dural sac. The intervertebral space and vertebral body lesion were accessed, and complete lesion debridement was performed using a bone knife or ultrasonic bone knife. Paravertebral abscesses were drained via a silicone tube.

###### 2.3.2.1.4 Lumbar tuberculosis

Pedicle screws were inserted, and the spinous process, lamina, and synovial joints on the severely affected side were excised. The dural sac was retracted medially to expose the anterior intervertebral space and vertebral body lesion, which was then debrided using a bone knife or ultrasonic bone knife.

###### 2.3.2.1.5 Intervertebral bone grafting

The nerve roots were retracted and protected, and the intervertebral height was measured. Autologous bone was harvested for intervertebral fusion. Instrumentation was applied with rods placed between the screws, and compression fixation was used for deformity correction. The procedures were confined to the diseased motion segment and did not involve adjacent healthy segments. Bone grafting was performed between the vertebral plates, articular processes, and transverse processes.

###### 2.3.2.1.6 Posterior-anterior combined approach

This approach combined the posterior and anterior techniques. Posterior internal fixation, deformity correction, and posterolateral intervertebral fusion were performed, followed by anterior lesion debridement, spinal canal decompression, and intervertebral fusion with autologous iliac bone grafts. The debridement steps were identical to the anterior-only approach.

##### 2.3.2.2 Non-intervertebral surgical approach

The anesthesia, surgical approach, lesion removal, decompression, deformity correction, and bone grafting techniques were identical to those used in the intervertebral group. However, internal fixation included long-segment fixation or single-segment fixation, extending beyond the diseased segment to include more than two normal motion units.

### 2.4 Postoperative management

Postoperatively, the vital signs of patients in both groups were closely monitored, with particular attention to the sensory and motor function of the lower extremities. The drainage tube was removed once the postoperative drainage volume was less than 20–50 mL. Patients were kept on strict bed rest for 4–6 weeks postoperatively and then allowed to mobilize with a brace. Functional exercises were encouraged during the bed rest period to prevent complications.

All patients continued with the preoperative triple or quadruple antituberculosis regimen, with adjustments made based on bacterial culture results or drug tolerance testing as needed. The duration of the treatment course was determined based on clinical manifestations, laboratory tests, and imaging evaluations during follow-up to assess tuberculosis cure.

### 2.5 Postoperative follow-up

Dedicated personnel were responsible for the follow-up and comprehensive management of anti-tuberculosis chemotherapy for all STB cases in our department. Postoperative follow-up was conducted once a month for the first 6 months, once every 6 months from 0.5 to 3 years, and once every 12 months thereafter. During each follow-up, patients’ medical history, laboratory findings, and imaging data were collected, and anti-tuberculosis medication guidance was provided. All data were systematically organized and archived.

### 2.6 Evaluation indicators

#### 2.6.1 Perioperative indicators

The perioperative indicators included the operation time, intraoperative blood loss, the need for blood transfusion, and the final visual analog scale (VAS) score for pain in both groups.

#### 2.6.2 Laboratory indicators

Laboratory indicators consisted of changes in erythrocyte sedimentation rate (ESR) and C-reactive protein (CRP) levels before surgery, at 6 months postoperatively, and at the final follow-up.

#### 2.6.3 Imaging indicators

The imaging indicators involved measuring the Cobb angle. The Cobb angle was defined as the angle between the extension line of the upper endplate of the normal vertebra adjacent to the diseased vertebra and the lower endplate of the next normal vertebra (positive values indicate anterior convexity, while negative values indicate posterior convexity).


$$\begin{array}{l} \mathrm{Correction rate}=(\mathrm{Preoperative kyphotic Cobb angle}-\\ \;\mathrm{Immediate postoperative kyphotic Cobb angle})\\ \;/\mathrm{Preoperative kyphotic Cobb angle}\times 100\% \end{array}$$

$$\begin{array}{l} \mathrm{Angle loss}=\mathrm{Final follow}-\mathrm{up}\;\mathrm{kyphotic Cobb angle}\\ \;-\mathrm{Immediate postoperative kyphotic Cobb angle} \end{array}$$

Bone graft healing was evaluated using computed tomography (CT) 3-dimensional (3D) reconstruction based on the following criteria: Presence of trabecular bone penetrating through the grafted area, forming a continuous bony bridge. (1) Fusion of the residual vertebral bone with the grafted bone. (2) Disappearance of gaps at the bone graft interface. (3) Neurological function recovery. Neurological function recovery was assessed using the Frankel classification at the preoperative stage and the final follow-up during the postoperative period.

### 2.7 Statistical analysis

Statistical analyses were performed using SPSS version 21.0 (IBM Corp., Armonk, NY, United States). Continuous variables were presented as mean ± standard deviation ( $\bar{x}\pm s$ ) or median (IQR), depending on data distribution. The independent *t*-test was used for normally distributed continuous variables, and the Mann–Whitney *U*-test for non-normally distributed data. Categorical variables were expressed as frequencies (%) and compared using the chi-square (*χ*^2^) test or Fisher’s exact test, as appropriate. A *p*-value <0.05 was considered statistically significant.

## 3 Results

### 3.1 Perioperative evaluation indices

The follow-up duration for all patients was complete and comprehensive. In Group A, the mean follow-up period was 63.24 ± 9.16 months, while in Group B, it was 64.05 ± 9.74 months. The perioperative evaluation revealed that the operation time, intraoperative blood loss, and the need for blood transfusion were significantly lower in Group A compared to Group B, with statistically significant differences (*p* < 0.05). At the last follow-up, patients in both groups reported no significant pain, and the VAS scores between the two groups showed no statistically significant difference (*p* > 0.05) (Table 4).

**Table 4:** Comparison of perioperative evaluation indexes between the two groups ( $\bar{x}\pm s$ ).

Observational indicators	Group A (*n* = 75)	Group B (*n* = 59)	Test value (*t*/*U*/*χ*^2^)	*p*
Follow-up duration (months)	63.24 ± 9.16	64.05 ± 9.74	−0.47	0.64
Operation time (mins)	193.79 ± 50.23	218.69 ± 56.78	−2.57	0.01
Amount of bleeding (mL)	817.33 ± 455.93	1111.86 ± 586.07	−3.11	<0.01
Blood transfusion or not (yes/no)	47/28	47/12	4.55	0.03
VAS score at the last follow-up	1.147 ± 0.833	0.780 ± 0.911	2.24	0.14

### 3.2 Laboratory examination indices

No statistically significant differences were observed in the ESR or CRP levels between the two groups during the preoperative period, 6 months postoperatively, and at the last follow-up (*p* > 0.05). Both ESR and CRP levels approached normal values by 6 months postoperatively and were within normal ranges by the final follow-up (Table 5).

**Table 5:** Comparison of ESR and CRP before surgery, 6 months after surgery and at the last follow-up between the two groups ( $\bar{x}\pm s$ ).

Group	*n*	Preoperative		6 months after surgery		Last follow-up	
		ESR (mm/h)	CRP (mg/L)	ESR (mm/h)	CRP (mg/L)	ESR (mm/h)	CRP (mg/L)
A	75	39.06 ± 25.07	29.34 ± 33.47	17.58 ± 13.14	4.90 ± 8.90	9.64 ± 8.32	1.21 ± 1.47
B	59	38.66 ± 23.39	26.71 ± 20.33	15.47 ± 13.67	3.30 ± 5.80	8.66 ± 8.91	1.59 ± 2.15
*t*		0.09	0.53	0.90	1.20	0.66	−1.22
*p*		0.93	0.597	0.37	0.23	0.51	0.23

### 3.3 Imaging evaluation indices

The Cobb angle showed no statistically significant differences between the two groups preoperatively, postoperatively, or at the final follow-up (*p* > 0.05). Similarly, there were no significant differences in the rate of Cobb angle correction or angle loss between the groups (*p* > 0.05). These findings indicate that intervertebral fixation of the affected vertebrae was equally effective in correcting deformities and restoring biomechanical stability of the spine in patients with MSTB (Table 6).

**Table 6:** Comparison of changes in cobb angle before surgery, 6 months after surgery, and at the last follow-up between the two groups ( $\bar{x}\pm s$ ).

Measurement period	Group A (n = 75)	Group B (n = 59)	*T*	*p*
Preoperative (°)	5.17 ± 20.06	3.49 ± 18.11	0.50	0.61
6 months after surgery (°)	10.86 ± 21.75	8.51 ± 18.46	0.66	0.50
Last follow-up (°)	10.19 ± 22.65	7.30 ± 21.78	0.74	0.45
Correction (°)	5.69 ± 10.12	5.02 ± 10.72	0.37	0.71
Lose (°)	−0.66 ± 3.40	−1.21 ± 8.59	0.49	0.62

Healing of the STB lesions, as evaluated through CT 3D reconstruction, demonstrated that the healing rate exceeded 88% at 6 months postoperatively and reached over 96% at 1 year postoperatively in both groups. At the final follow-up, the implants were fully healed, with no statistically significant differences between the two groups (*p* > 0.05) (Table 7).

**Table 7:** Comparison of bone graft healing between the two groups.

Group	*n*	6 months after surgery	1 year after surgery	Last follow-up
A	75	66 (88.00%)	73 (97.33%)	75 (100%)
B	59	53 (89.83%)	58 (98.31%)	59 (100%)
*χ* ^2^		0.11	0.07	0.00
*p*		0.74	0.79	1.00

**Figure 2:**
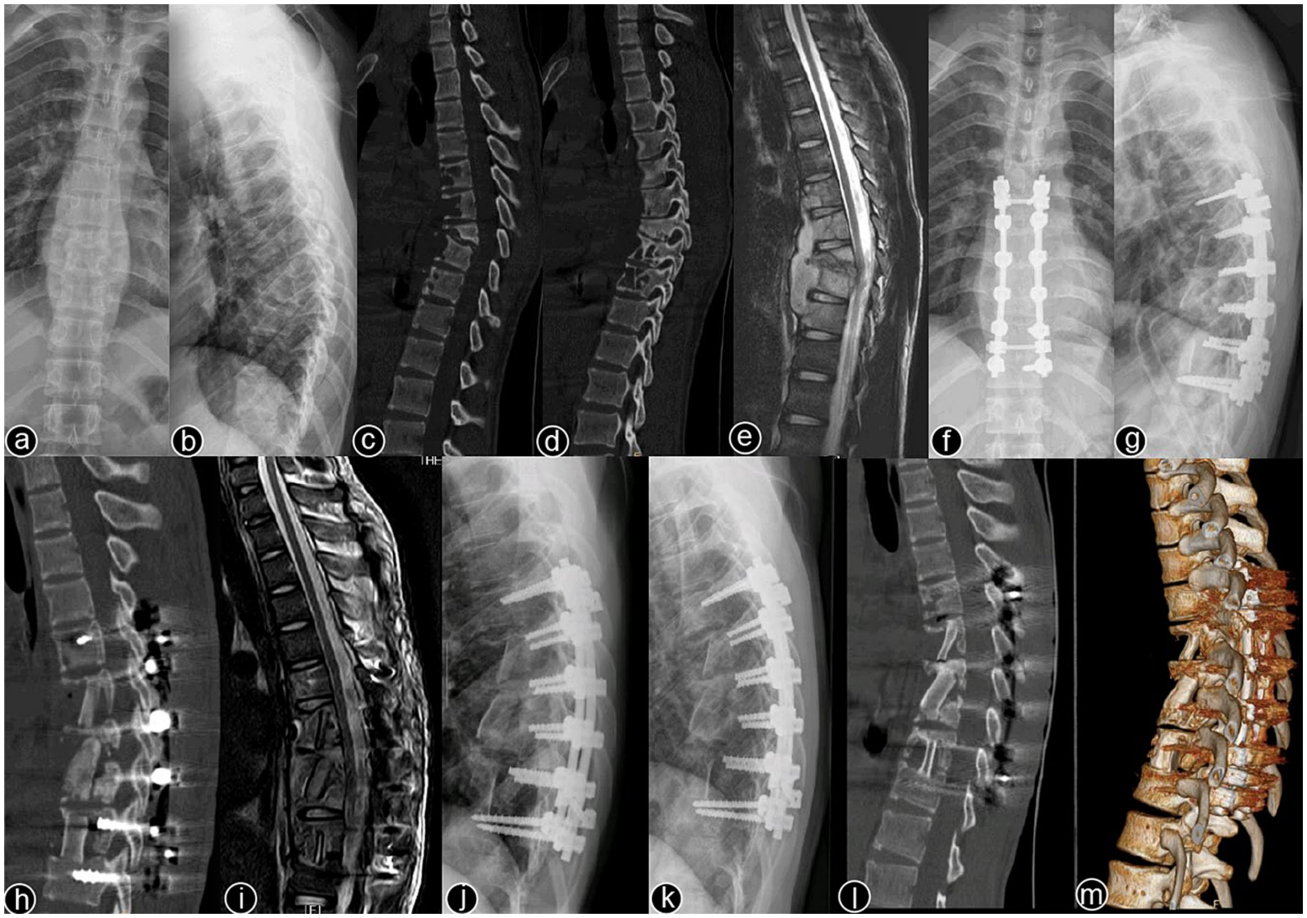
Patient, male, 51 years old. Diagnosis: spinal tuberculosis (T6–T11). Surgical approach: intervertebral surgery. **(A,B)** Preoperative anteroposterior and lateral X-rays of the thoracic spine show roughened anterior margins of the T6–T11 vertebrae, narrowed intervertebral spaces, and kyphotic deformity. **(C,D)** Preoperative thoracic spine CT reveals narrowing of the intervertebral spaces, destruction of multiple vertebral bodies, paravertebral soft tissue swelling, and significant vertebral collapse with kyphotic deformity. **(E)** Preoperative thoracic spine MRI shows multiple abnormal vertebral signals, destruction of vertebral bodies and intervertebral spaces, and the formation of paravertebral abscesses. **(F–I)** Immediate postoperative anteroposterior and lateral X-rays, CT, and MRI of the thoracic spine show short-screw fixation at T7–T10, complete lesion removal, and satisfactory bone graft placement. **(J)** One-month postoperative lateral X-ray shows good internal fixation position with effective correction of the kyphotic deformity. **(K–M)** One-year postoperative lateral X-rays, CT, and 3D reconstruction show no loosening or breakage of the internal fixation, good bone graft positioning, and blurred bone graft interfaces, indicating healing.

**Figure 3:**
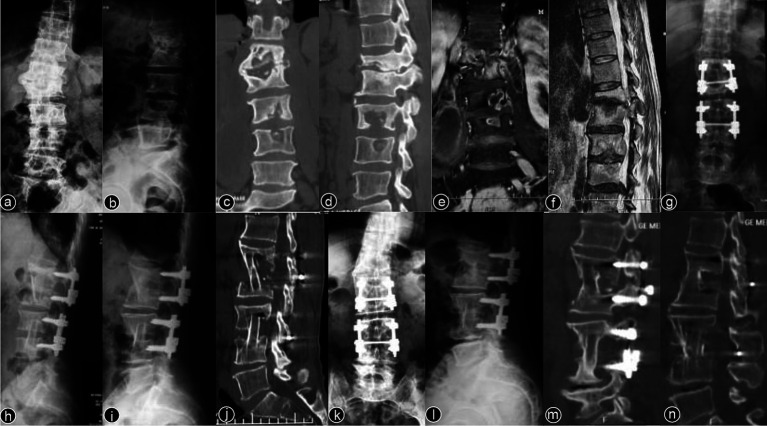
Patient, male, 38 years old. Diagnosis: spinal tuberculosis (L1–L2/L3–L4). Surgical approach: intervertebral surgery. **(A,B)** Preoperative anteroposterior and lateral X-rays of the lumbar spine show roughened anterior margins of the L1–L4 vertebrae and narrowing of the intervertebral spaces. **(C,D)** Preoperative lumbar CT reveals narrowing of the L1–L2/L3–L4 intervertebral spaces, multiple non-contiguous vertebral body destruction, and scoliosis. **(E,F)** Preoperative lumbar MRI demonstrates narrowing of the L1–L2/L3–L4 intervertebral spaces, abnormal vertebral signals, and an epidural abscess occupying the spinal canal. **(G,H)** Immediate postoperative anteroposterior and lateral X-rays of the lumbar spine. **(I,J)** Two-year postoperative lateral X-rays and CT scans show stable internal fixation and well-healed bone grafts. **(K–N)** Ten-year postoperative anteroposterior and lateral X-rays and CT scans confirm no loosening or breakage of the internal fixation, with solid bone graft fusion and no recurrence of the local lesion.

**Figure 4:**
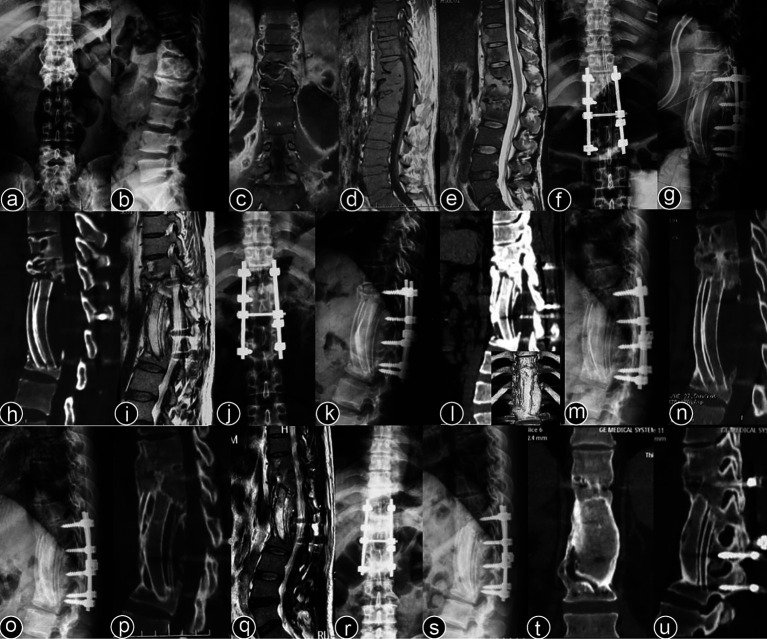
Patient, male, 45 years old. Diagnosis: spinal tuberculosis (T11–L2). Surgical approach: intervertebral surgery. **(A,B)** Preoperative anteroposterior and lateral X-rays of the thoracic spine show narrowing of the T11–L2 intervertebral space, significant vertebral destruction, and kyphotic deformity. **(C–E)** Preoperative thoracolumbar MRI reveals multiple vertebral and accessory signal changes, narrowing of the intervertebral spaces, and a large paravertebral abscess. **(F–I)** One-month postoperative anteroposterior and lateral X-rays, CT, and MRI of the thoracolumbar spine show short-screw fixation at T11–L2, with proper rib graft placement at the defected segments and a clear spinal canal. **(J–L)** One-year postoperative anteroposterior and lateral X-rays and CT scans show good fixation with solid bone graft fusion. **(M,N)** Two-year postoperative lateral X-rays and CT scans. **(O–Q)** Five-year postoperative lateral X-rays, CT, and MRI of the thoracolumbar spine. **(R–U)** Ten-year postoperative lateral X-rays and CT scans show stable internal fixation without loosening or breakage, solid bone graft fusion, and no recurrence of the local lesion.

**Figure 5:**
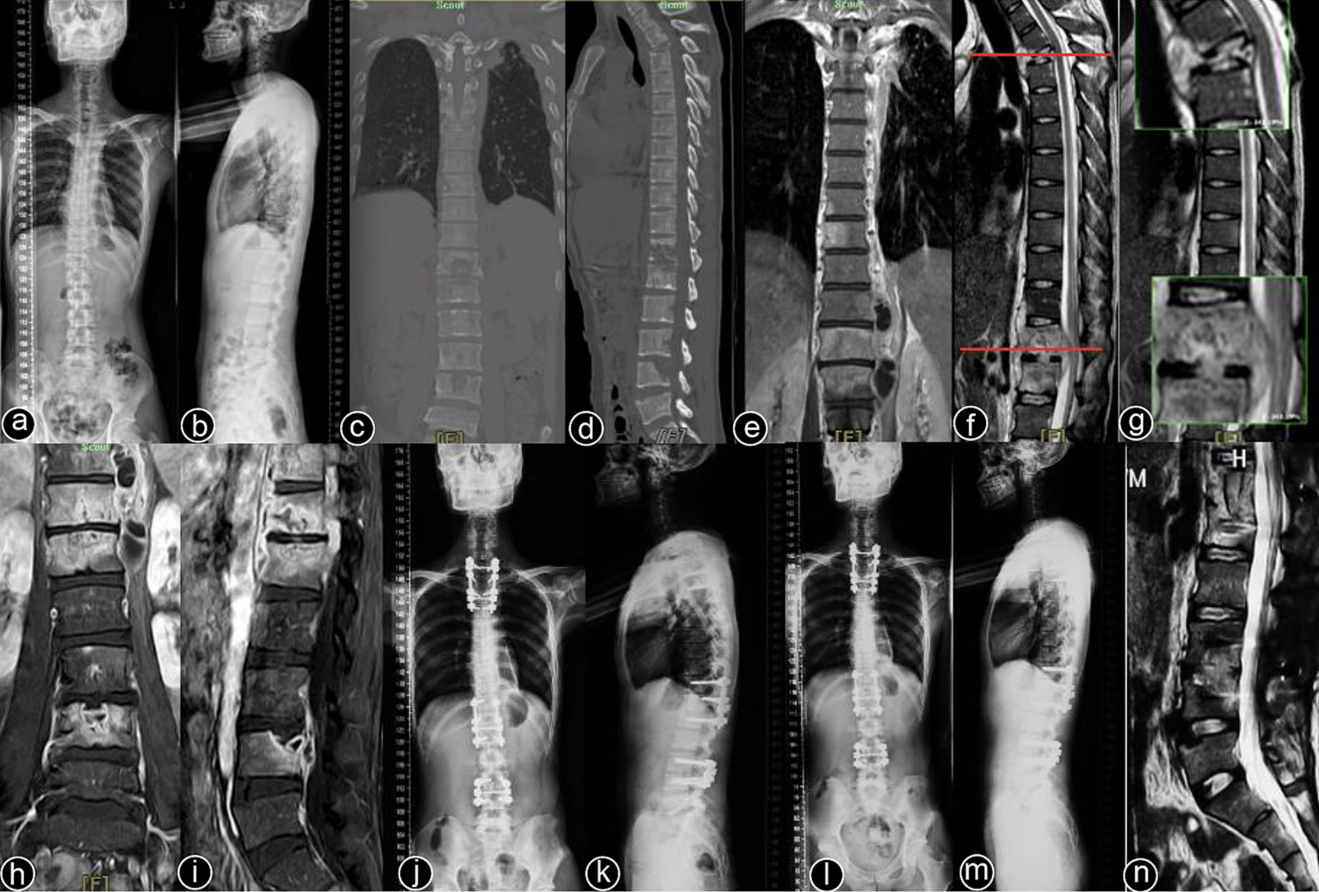
Patient, male, 48 years old. Diagnosis: spinal tuberculosis (T3, T12–L1, L3–L4). Surgical approach: non-intervertebral surgery at T1–T4 and T11–L1; intervertebral surgery at L3–L4. **(A,B)** Preoperative full-spine anteroposterior and lateral X-rays show unclear visualization of the T3 vertebral body, with roughened anterior margins and narrowed intervertebral spaces at T12–L1 and L3–L4. **(C,D)** Preoperative full-spine CT reveals multiple non-contiguous vertebral body destructions with paravertebral soft tissue swelling. **(E–I)** Preoperative thoracic and lumbar MRI show abnormal signal intensities in multiple thoracic and lumbar vertebrae, with narrowed intervertebral spaces and extensive paravertebral abscess formation. **(J,K)** Immediate postoperative full-spine anteroposterior and lateral X-rays show stable internal fixation at T1–T4, T12–L1, and L3–4. **(L–N)** One-year postoperative full-spine anteroposterior and lateral X-rays, and lumbar MRI show no loosening or breakage of the internal fixation, a clear spinal canal, and no recurrence of the local lesion.

**Figure 6:**
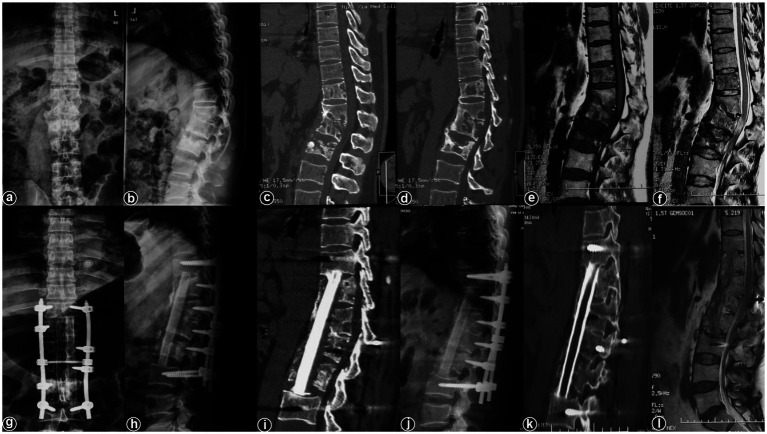
Patient, female, 45 years old. Diagnosis: spinal tuberculosis (T11–L2). Surgical approach: non-intervertebral surgery. **(A,B)** Preoperative anteroposterior and lateral X-rays of the thoracic spine show narrowing of the T11–L2 intervertebral space and kyphotic deformity. **(C,D)** Preoperative thoracic CT shows narrowing of the intervertebral spaces and destruction of multiple vertebral bodies. **(E,F)** Preoperative thoracic MRI reveals multiple abnormal vertebral signals, narrowing of the intervertebral spaces, and spinal cord compression. **(G–I)** Immediate postoperative anteroposterior and lateral X-rays, and CT of the thoracic spine show short-screw fixation at T11–L2, proper removal of the intervertebral lesion, and satisfactory bone graft placement. **(J–L)** Three-year postoperative lateral X-rays, CT, and MRI of the thoracic spine show stable internal fixation without loosening or breakage, good bone graft healing, a clear spinal canal, and no recurrence of the local lesion.

Typical cases are shown in Figures 2–6, with Figures 2–4 illustrating intervertebral fixation of the affected vertebrae in three patients from Group A, and Figures 5, 6 demonstrating non-intervertebral fixation in two patients from Group B.

### 3.4 Postoperative neurological function recovery

Neurological function improved significantly in both groups at the last follow-up compared to the preoperative period. Significant recovery of neurological deficits was observed in both groups, as shown in Table 8.

**Table 8:** Frankel grading of neurological function in the two groups before surgery and at the last follow-up.

Group	*n*	Grading	Preoperative	Last follow-up		
				C	D	E
A	75	B	1	1		
		C	10		1	9
		D	22		5	17
		E	42			42
B	59	B	2	1	1	
		C	17		3	14
		D	30		3	27
		E	10			10

### 3.5 Complications

No severe neurological complications, including paraplegia or cauda equina syndrome, were observed in either group. In Group A, three patients experienced wound complications such as infection, fat liquefaction, and delayed wound healing, all of which were effectively managed through dressing changes, debridement, and antimicrobial therapy. Two patients in Group A developed tilted bone grafts, which were successfully treated with enhanced anti-tuberculosis therapy and graft stabilization. Additionally, seven patients in this group experienced anti-tuberculosis drug-related complications, managed by adjusting the medication regimen.

In Group B, two patients experienced bone graft resorption and pedicle screw loosening, while two others developed recurrent lumbar psoas abscesses, which were successfully treated with reoperation and intensified anti-tuberculosis chemotherapy. Furthermore, two patients had wound infections, which resolved with medication adjustments and antimicrobial therapy. Four patients in Group B had complications related to anti-tuberculosis medications, addressed through appropriate modifications to their treatment plans.

## 4 Discussion

STB is the most common form of bone and joint tuberculosis. In regions where tuberculosis is endemic, many STB patients fail to receive timely and effective diagnosis and treatment due to the insidious onset of the disease, leading to involvement of multiple spinal segments (12, 13). MSTB can be classified into continuous and discontinuous forms. Previous studies have reported the incidence of MSTB to range from 2.1 to 5% (14). However, in our study, the incidence of MSTB was found to be 12.45%, which is significantly higher than that reported in previous research. This higher incidence may be closely related to the endemic nature of tuberculosis in this region. Continuous MSTB notably increases the risk of spinal instability, kyphotic deformity, and paraplegia (15). Nevertheless, research on the clinical characteristics of MSTB remains limited. In our study, the age of onset ranged from 11 to 78 years, with a higher incidence among farmers. Additionally, 50% of the patients had a confirmed history of pulmonary tuberculosis, and all patients presented with severe systemic tuberculosis toxicity. Local mechanical instability resulting in pain was observed in 94.78% of the cases, CRP levels were markedly elevated in 95.52%, and kyphotic deformity was noted in 63.43%. Furthermore, more than 90% of the patients exhibited abscess formation at various sites. Compared to other forms of STB, MSTB—particularly the discontinuous form—can be difficult to differentiate from conditions such as lymphoma, multiple myeloma, metastatic tumors, osteoporotic fractures, or other spinal infections (16). Vertebral destruction leading to spinal instability, abscess formation, and sequestrum formation are the primary causes of spinal cord or nerve dysfunction in MSTB, and these complications are among the most severe (17).

MSTB is a relatively rare but highly severe condition (18). While anti-tuberculosis (TB) pharmacotherapy remains the cornerstone of STB treatment (19), early surgical intervention, including lesion debridement, spinal cord decompression, and stabilization, is crucial in reducing the risk of spinal cord dysfunction, progressive spinal instability, and deformity. It also improves cure rates, accelerates recovery, and shortens treatment duration (20). Despite the established role of anti-TB therapy, surgery is essential for patients with large paravertebral abscesses, spinal cord or nerve compression, spinal instability, or severe kyphotic deformity (21). However, prospective, multicenter, large-scale randomized controlled trials on comprehensive management for STB are limited, and studies specifically focusing on MSTB are particularly scarce. The treatment principles for MSTB generally mirror those for single-segment STB, but personalized treatment plans are necessary. In our study, clinical and imaging analyses of 134 MSTB cases indicated absolute or relative indications for surgery in all patients. Postoperative cure rates exceeded 95% after 1 year in both groups, suggesting that surgery is an effective treatment strategy for MSTB (22). For anti-TB pharmacotherapy, the World Health Organization (WHO) guidelines for pulmonary TB treatment emphasize combination therapy, correct dosages, regular administration, and full-course completion to minimize recurrence. However, the optimal regimen and duration for STB treatment remain inconclusive. Typically, current regimens include an initial four-drug combination phase (isoniazid, rifampin, pyrazinamide, and ethambutol), followed by a continuation phase with a three-drug regimen (isoniazid, rifampin, and ethambutol). WHO recommends at least 6 months of treatment for bone and joint TB (23), while Indian guidelines suggest 10–16 months (24), and Chinese expert consensus recommends 12–18 months (25). Our team, led by Wang et al. (26, 27), proposed a short-course chemotherapy regimen (2SHRZ/2–4HRZ) in 2007 for STB, with favorable outcomes, and a treatment duration of 4–6 months, averaging 4.5 months. However, few studies have explored the applicability of short-course chemotherapy for MSTB, possibly due to its low incidence and complex treatment needs. In our study, 52.99% (71 cases) of patients received standard chemotherapy, while 47.01% (63 cases) received short-course chemotherapy. Both regimens achieved clinical cure when combined with thorough lesion debridement.

The primary goal of surgical treatment for MSTB is to eradicate TB lesions, relieve spinal cord or nerve compression, reconstruct spinal integrity and stability, and prevent or correct spinal deformities. Studies by Gillet (28) and Sudo et al. (29) have demonstrated that longer fixation segments result in greater stress concentration and increased forces on adjacent segments, which in turn accelerate the degeneration of these adjacent segments. To overcome the disadvantages associated with long-segment and short-segment fixation, we aimed to reduce the scope of internal fixation, thereby avoiding the accelerated degeneration of adjacent segments and the loss of spinal mobility caused by excessive fixation. All surgical procedures were confined to the vertebral bodies affected by *Mycobacterium tuberculosis* without involving the adjacent healthy vertebrae (9). Our team, led by Professor Shi, reported satisfactory clinical outcomes with Intervertebral focal surgery for non-contiguous MSTB in 2012 (11). In this study, we conducted a more in-depth investigation that was not limited to non-contiguous MSTB and expanded the sample size to enhance the level of evidence. The operation time, amount of bleeding, and blood transfusion or not in the intervertebral surgery group were significantly better than those in the non-intervertebral surgery group, with statistically significant differences. There were no significant differences between the two groups in preoperative, postoperative, or final follow-up Cobb angles, correction rates, or angle loss (*p* > 0.05). Neurological function in both groups significantly improved at the final follow-up compared to preoperative status. Liu et al. (30) suggested that posterior-only debridement, interbody fusion, and long-segment fixation might be a viable surgical option for MSTB in the thoracic spine. However, long-segment fixation in the lumbar spine inevitably affects functional motion units. Unfortunately, in this study, the incidence of adjacent segment disease (ASD) in patients undergoing long-segment fixation in Group B has not been fully analyzed. Regarding the choice of surgical approach, Zhong et al. (31) proposed that a single-stage posterior approach is a feasible option for consecutive multisegmental thoracic TB with kyphosis, as it allows for simultaneous debridement, decompression, and stabilization. Li et al. (32) demonstrated that all three approaches—anterior-only, posterior-only, and combined anterior-posterior—can achieve safe and effective neural decompression, graft fusion, and kyphosis correction. The selection of the surgical approach should be tailored to the patient’s overall condition, lesion characteristics, comorbidities, and the surgeon’s expertise, with an emphasis on minimizing surgical trauma and facilitating rapid recovery. This aligns with our perspective.

There are limited reports in the literature regarding the optimal surgical approach for MSTB. Based on our experience, we propose the following fundamental principles for selecting the appropriate surgical approach for MSTB: the primary objectives should be the complete removal of tuberculous lesions, relief of neurological and spinal cord compression, restoration of spinal stability, and correction of spinal deformity. Surgeons should select the approach with which they are most familiar and proficient, ensuring that the procedure is performed with the highest level of expertise. Additionally, it is critical to minimize surgical trauma to the patient to preserve their overall health and well-being. This approach aligns with the modern surgical concept of enhancing rapid recovery. In the present study, we performed diseased interbody fixation and fusion as well as non-diseased interbody fixation and fusion in patients with MSTB. The results demonstrated that diseased interbody fixation and fusion achieved comparable outcomes in deformity correction and implant fusion, with some clinical indicators showing superior results in the diseased interbody fixation group compared to the non-diseased interbody fixation group. While diseased intervertebral fixation has achieved favorable clinical outcomes in MSTB, it is crucial to strictly define and adhere to the indications and contraindications for its clinical application. The guiding principle for diseased intervertebral surgery is: “Extent of the lesion determines the extent of debridement; extent of debridement determines the extent of fusion; extent of fusion determines the extent of fixation (11).” In essence, the surgical intervention should be limited to the affected vertebrae and intervertebral discs without sacrificing or damaging healthy vertebrae and discs. Based on our research findings and clinical experience, the indications for diseased intervertebral surgery are as follows (9): (1) the pedicle is either unaffected or only mildly compromised by the tuberculous lesion, providing at least 60% of the pedicle screw’s pull-out strength; (2) after debridement, the upper and lower endplates of the affected vertebrae should remain intact, providing a stable bed for bone grafting; (3) there is no significant osteoporosis.

This study demonstrated favorable clinical outcomes; however, several limitations must be acknowledged. As a retrospective, single-center case–control study, the sample size was relatively small due to the low incidence of MSTB. Consequently, further validation through multicenter, randomized controlled trials with larger cohorts is necessary to strengthen the evidence base and improve the generalizability of the findings.

## 5 Conclusion

Under strict adherence to surgical indications, intervertebral surgery for MSTB is shown to be safe, effective, and feasible and may represent a viable option for managing these patients.

## Data Availability

The raw data supporting the conclusions of this article will be made available by the authors, without undue reservation.
